# Tissue Fibrosis Decoded *via* Cellular Senescence: Mechanisms, Treatments, and Emerging Technologies

**DOI:** 10.14336/AD.2025.0503

**Published:** 2026-07-18

**Authors:** Wenjie Cai, Haoyu Zhang, Zhouzhou Li, Mingyun Cai, Shiwen Yu, Peng Chen, Xinyu Song

**Affiliations:** ^1^Department of Respiratory and Critical Care Medicine, The first College of Clinical Medicine science, China Three Gorges University, Yichang, China.; ^2^Department of Clinical Medical Research Center for Precision Diagnosis and Treatment of Lung Cancer and Management of Advanced Cancer Pain of Hubei Province, Yichang, China.; ^3^Department of Respiratory and Critical Care Medicine, Yichang Central People's Hospital, Yichang, China.; ^4^Experimental Research Center, China Academy of Chinese Medical Sciences, Beijing, China.

**Keywords:** E fibrosis, senescence, senolytics, multi-omics techniques

## Abstract

Cellular senescence is one of the hallmarks of aging, and it is closely related to tissue fibrosis in various age-related diseases. By integrating multi-level and multi-dimensional data, emerging technologies can comprehensively analyze the complex mechanisms of aging and fibrosis diseases, discover new biomarkers and therapeutic targets, and promote the development of precision medicine. Most of the current reviews still focus on the molecular mechanisms of cellular senescence and fibrosis diseases, but lack a comprehensive summary of anti-aging drugs for the treatment of fibrosis-related diseases, and emerging technologies and integrated analysis of multi-omics data. This review discusses the latest evidence on the role of cellular senescence in tissue fibrosis, especially lungs, liver, and kidney fibrosis. In addition, we also introduce the application of anti-aging strategies in fibrotic diseases, especially drugs to eliminate senescent cells, and the latest application of emerging technologies in aging and fibrotic diseases.

## 1. Introduction

Aging is a universal process that occurs all the time [1, 2]. Cell senescence is characterized by genomic instability [3, 4], telomere attrition and dysfunction [5-7], changes in gene epigenetics, decreased autophagy [8, 9], mitochondrial dysfunction, Senescence Associated Secretory Phenotype (SASP) [10, 11], endoplasmic reticulum stress, and stem cell dysfunction. It can lead to stem cell and parenchymal cell dysfunction to reduce tissue resistance to pathogenic stress. At present, the most studied SASP secreted by senescent cells is closely related to aging [20]. The inflammatory response caused by SASP plays an important role in the disease [21], and SASP mediated increase in extracellular matrix is a key factor in disease progression or inhibition. SASP can also affect normal cell physiological function and promote normal cell aging through autocrine or paracrine forms [22, 23].

Many diseases have been reported to be related to cell aging, such as degenerative diseases such as Alzheimer's disease, Parkinson's disease [24], arthritis [25], metabolic diseases such as diabetes, atherosclerosis [26], cancer [27, 28], fibrosis and other related diseases [24-28]. Ballinger [33] et al. found and confirmed that mitochondrial dysfunction can cause atherosclerotic lesions and atherosclerotic damage. The proinflammatory factors contained in SASP induce epithelial-mesenchymal transition and invasion, which are the hallmark characteristics of malignant tumors [36]. The chronic inflammatory response caused by SASP enhances the local inflammatory response of microglia, which is an important pathological change of Alzheimer's disease. At the same time, the metalloproteinase (MMP3) in SASP leads to the abnormal differentiation of epithelial cells into fibroblasts. The secretion of Vascular Endothelial Growth Factor (VEGF) by senescent fibroblasts can lead to abnormal proliferation of vascular endothelial cells and then lead to atherosclerosis [37]. Chronic inflammation caused by mutations in specific genes (such as ERCC1-XFP) [38], abnormal autophagy function, and stress response [39] promotes cellular senescence and accelerates the disease process.

**Figure 1.**
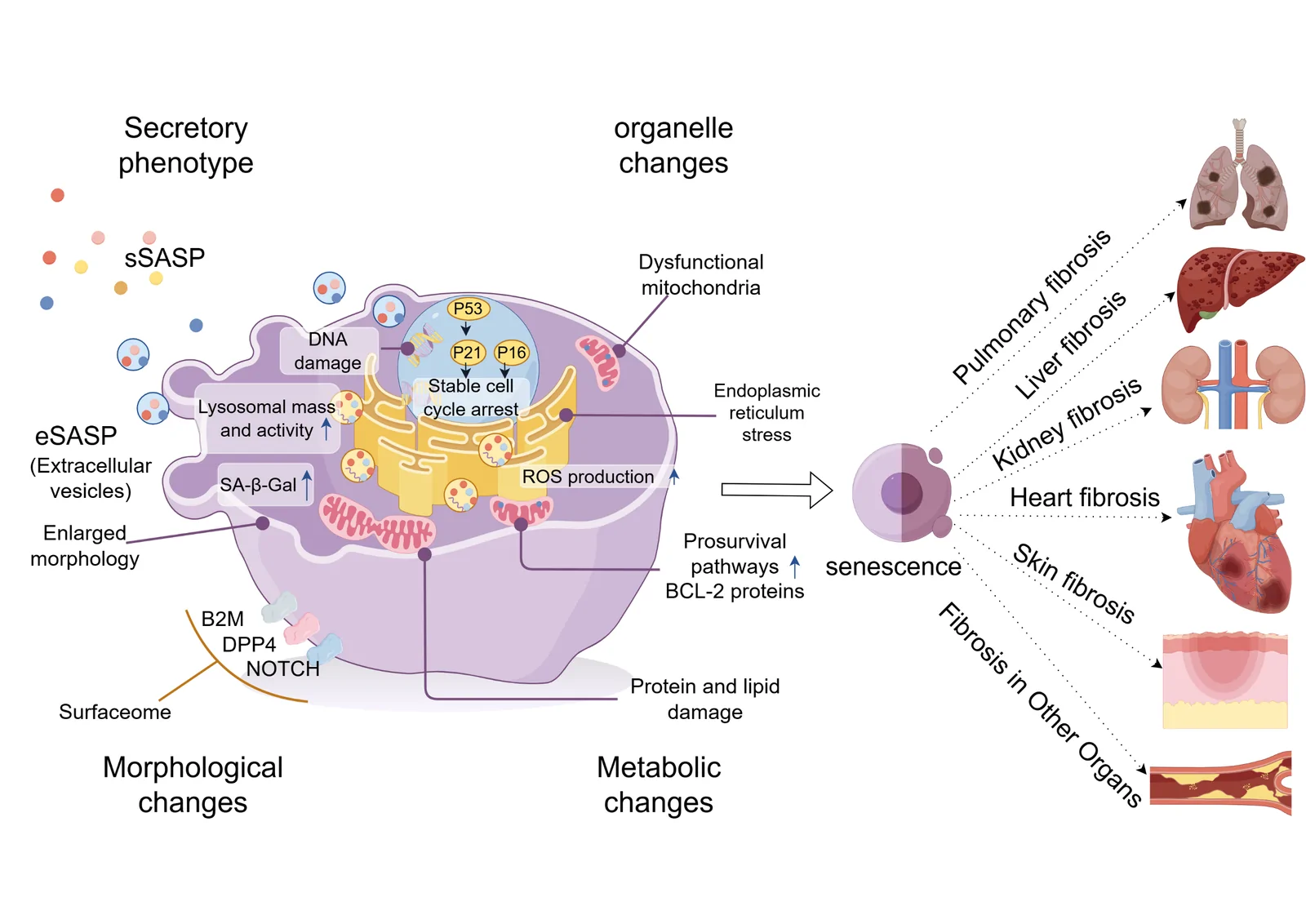
**Senescence induced cellular macromolecular alterations and their contribution to tissue fibrosis**. Senescent cells secrete senescence-associated secretory phenotypes that elicit a series of intracellular responses. Senescent cells are also accompanied by different degrees of organelle changes, such as endoplasmic reticulum stress, mitochondrial dysfunction, and DNA damage. At the same time, the morphology of senescent cells itself changes: cells lose their original shape and become round and larger.

Fibrosis is both a dynamic developmental process and a final pathological state. When the body is subjected to chronic and persistent injury, the imbalance between fibrotic and anti-fibrotic homeostasis in the body leads to collagen deposition, permanent scar formation, organ remodeling and dysfunction, and eventually death [40]. More and more studies have proved that cellular senescence is closely related to fibrotic diseases. "In age-related fibrotic diseases such as idiopathic pulmonary fibrosis (IPF), the accumulation of profibrotic senescent fibroblasts leads to progressive fibrosis [264, 41-43]. As one of the most important characteristics of cellular senescence [41], SASP contains proinflammatory mediators such as interleukin-6(IL-6), epidermal growth factor receptor (TGF-β) and other growth factors, by activating fibroblasts to differentiate into myofibroblasts and promoting excessive deposition of extracellular matrix (ECM), it participates in the process of tissue fibrosis [47, 42]. Senescent parenchymal cells (such as alveolar epithelial cells, hepatocytes, and renal tubular epithelial cells) release reactive oxygen species (ROS) and profibrotic factors through paracrine effects, altering the local microenvironment and inducing the activation of adjacent interstitial cells (such as fibroblasts and stellate cells), thus forming a fibrotic feedforward circuit [43]. Meanwhile, fibrosis leads to microcirculation disorders, local hypoxia and ROS accumulation, promoting cell senescence by activating the p53/p21 pathway, thereby forming a vicious cycle of senescent cell accumulation [44].

The rapid development of multi-omics emerging technologies such as single-cell sequencing and organoid chips has continuously confirmed the close relationship between cellular senescence and fibrosis. Moreover, emerging multi-omics technologies have played an irreplaceable role in early disease diagnosis and prediction of disease progression, and continue to find anti-aging strategies to provide new treatment methods for anti-fibrosis. With the application of anti-aging methods in the treatment of organ fibrosis, the close relationship between aging and fibrosis has been confirmed [45-48]. This article summarizes the different characteristics of cellular senescence and the molecular mechanism of fibrosis and briefly reviews the application of anti-aging strategies in the treatment of fibrosis-related diseases and multi-omics emerging technologies in aging and fibrosis (Fig. 1).

## 2. The molecular mechanistic link between cellular aging and tissue fibrosis

### 2.1 Telomere dysfunction and fibrosis

Mutations in the genes encoding telomerase reverse transcriptase (TERT) and telomerase RNA are the most common causes of telomere shortening and dysfunction [50]. TERT gene mutations in familial IPF can lead to alveolar stem cell failure and aggravate pulmonary fibrosis [51, 52]. In addition, telomere attrition and DNA damage have also been confirmed to activate the P53 pathway to inhibit mitophagy [53], upregulate endoplasmic reticulum stress markers, intensify the accumulation of ECM and senescent mitochondria in lung tissue, and then activate fibroblasts for proliferation and differentiation [54, 55]. Povedano et al. successfully alleviated the process of pulmonary fibrosis by transgenic expression of TERT, which became a potential therapeutic strategy for IPF [56]. Telomere dysfunction in primary sclerosing cholangitis can promote the senescence of cholangiocytes and biliary fibrosis [57]. In a Mendelian randomization study [58], it was confirmed that the shorter the telomere in leukocytes, the higher the risk of liver fibrosis, and a number of studies and clinical cases [59-62] have shown that telomere shortening and dysfunction in hepatocytes and leukocytes promote cellular senescence, and activate SASP secretion by hepatic stellate cells to promote liver fibrosis. Restoration of hepatic telomerase activity leads to a reduction in cirrhosis and improvement in liver function [63].

**Figure 2.**
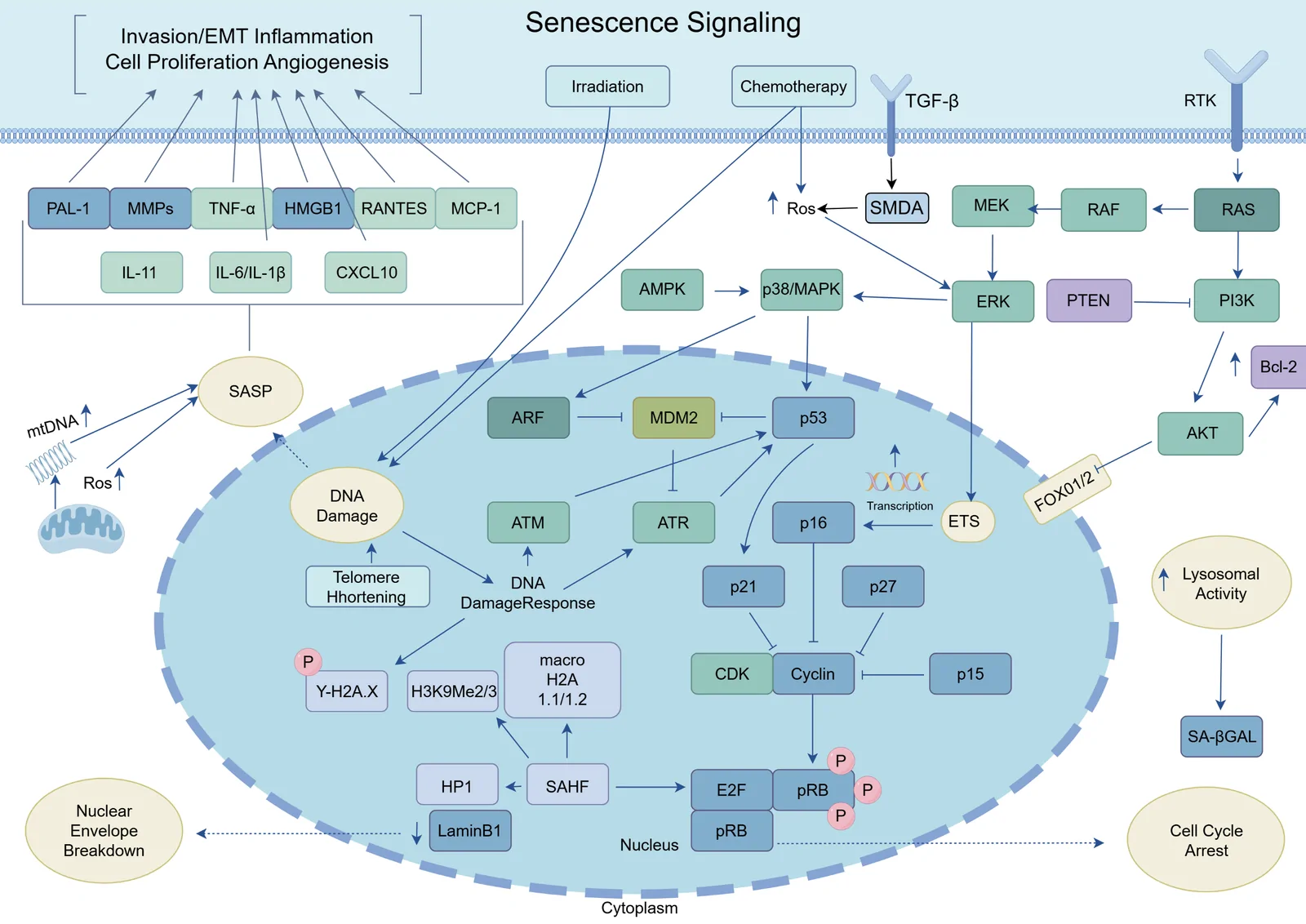
**Classic molecular mechanisms of cellular senescence leading to tissue fibrosis**. A variety of inflammatory factors contained in SASP act on the surface receptors of normal cell membrane, activate senescence and verify related pathways, cyclin P53 is increased, P21, P16 related senescence proteins are also increased, leading to normal cell senescence. In addition, mitochondrial dysfunction in senescent cells leads to increased reactive oxygen species, cell cycle arrest, telomere wear, and DNA damage, which can further promote SASP secretion in senescent cells and cause tissue fibrosis.

Moreover, telomere wear and dysfunction can activate telomeric silencing interferon-like1 (Dot1L) in telomeres. Activated Dot1L increases the level of ROS through PI3K-AKT signaling, accelerates renal tubular epithelial cell senescence and promotes renal parenchymal fibrosis [65, 66]. In many studies, intervention measures based on telomerase and telomerase gene in the process of fibrosis not only improve fibrosis, but also prolong life span, providing more and more potential therapeutic strategies for the treatment of fibrosis [67, 68].

Taken together, telomere dysfunction promotes cell senescence, which promotes cell senescence and progression of fibrosis-related diseases. There is evidence that when telomerase is genetically reactivated, it can delay normal aging in mice and even reverse premature aging in telomerase-deficient mice [69]. Therefore, regulating cell senescence through telomerase activation can delay the progression of fibrosis-related diseases, which is also a new therapeutic strategy (Fig 2).

### 2.2 Senescence-Associated Secretory Phenotype and fibrosis

Senescent cells secrete SASP in different forms such as autocrine and paracrine to change the microenvironment [70]. TGF-β, VEGF, IL-6 and chemokines such as CCL2 contained in SASP can not only transmit aging information to neighboring cells in the form of paracrine, but also accelerate their own aging in the form of autocrine. Moreover, it can activate fibrocytes to varying degrees and promote fibrosis [71]. When tissue is damaged, SASP can promote neighboring cells to undergo cellular reprogramming [72] and enhance plasticity [73]. Long-term chronic injury stimulation, continuous cell reprogramming and tissue and organ remodeling, tissue and organ elasticity changes into irreversible fibrosis, leading to organ dysfunction. In addition, SASP can also induce Epithelial-to-Mesenchymal Transition (EMT), which leads to fibrosis [74, 75].

**Figure 3.**
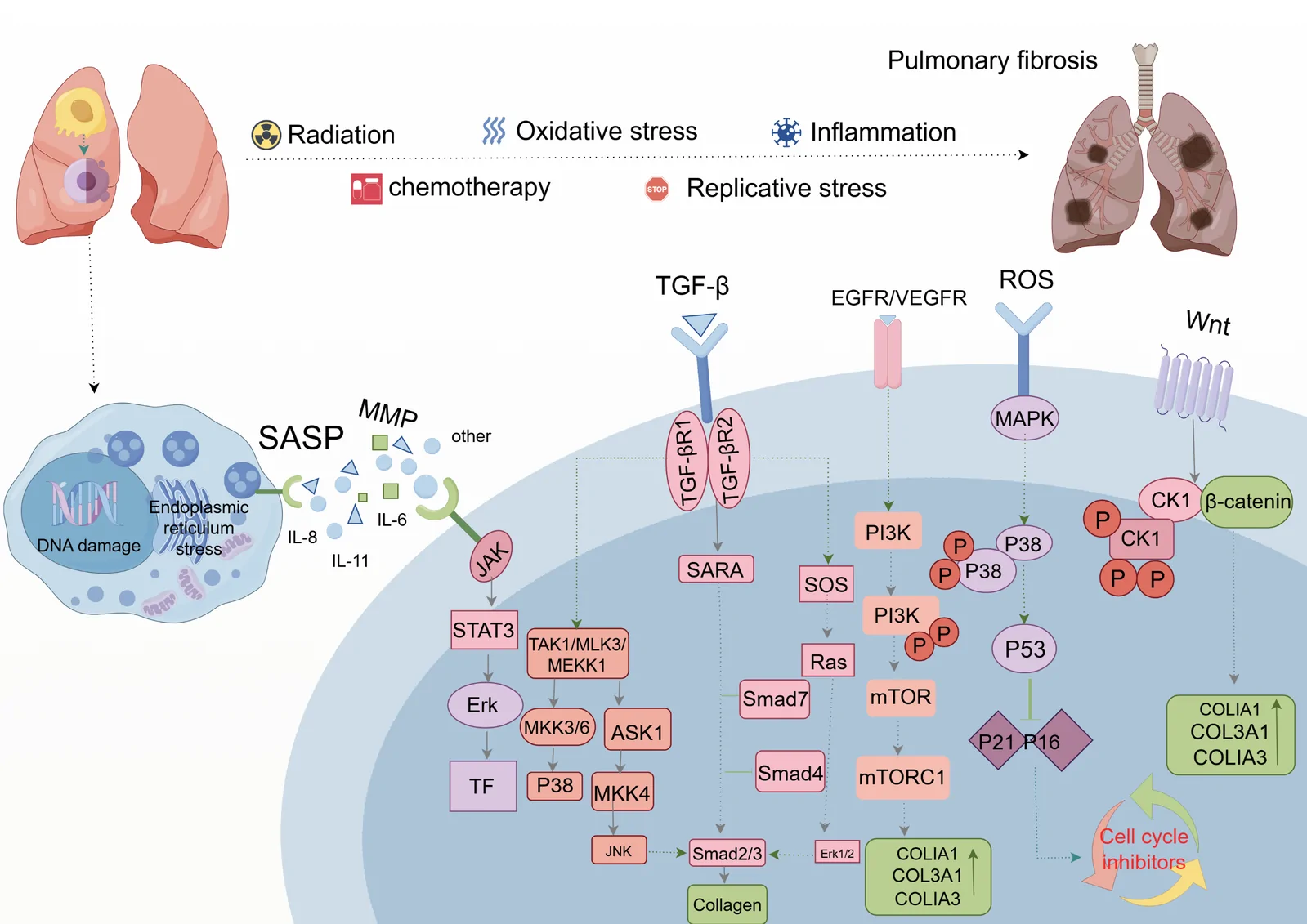
**Diagram of the molecular mechanism of SASP leading to pulmonary fibrosis**. When the lung is subjected to external stimuli such as radiation, chemical injury, oxidative stress, etc., senescent cells release SASP through paracrine or autocrine forms. The inflammatory factors and pro-fibrotic molecules contained in SASP act on normal or senescent cells to activate pro-fibrotic pathways such as TGF-β, WNT, JAK and other related pathways. The increase of P53, P21, and P16 leads to cell cycle arrest, while the increase of collagen promotes the process of fibrosis.

IL-8 in SASP can cause renal tubular cell cycle arrest, repeated and prolonged renal parenchyma inflammation, and then activate myofibroblasts, increase and accumulate ECM, eventually leading to progressive chronic renal fibrosis [76, 77]. In damaged alveoli, alveolar epithelial cell senescence not only affects the repair and regeneration of normal alveolar cells, but also induces healthy cell senescence by changing the alveolar microenvironment through SASP paracrine effect [78], activates TGF-β and Wnt signaling pathways [79], and promotes the differentiation and proliferation of fibroblasts into myofibroblasts [80]. At the same time, more SASPs can promote cell senescence and form a chronic persistent inflammatory state through different cell communication cascades. SASP secreted by senescent alveolar epithelial cells can make immune cells dysregulated [81], further block the repair of alveolar cells, especially type II alveolar epithelial cells (AT2) [82, 83], and promote the process of fibrosis [84]. In addition, fibrosis scar caused by hepatocyte senescence in liver cirrhosis has been used as one of the markers of liver cirrhosis [85], and hepatocyte senescence is positively correlated with liver fibrosis stage. CCL2 in SASP can stimulate liver macrophages to produce TGF-β factor, activate hepatic stellate cells, and promote liver fibrosis [86]. The potential harm of senescent cholangiocytes in biliary tract diseases has also been reported in detail by establishing K19-Cre-Mdm2 f/f in vivo models [87, 88]. Autonomic senescence of cholangiocytes leads to the transformation of adjacent normal cells into involuntary senescence in the form of paracrine. P53-induced senescent hepatic stellate cells proliferate and secrete a large amount of ECM, leading to EMT and intrahepatic biliary sclerosis [89]. Researchers extracted SASP from systemic sclerosis fibroblasts taken and added to normal healthy fibroblast samples. They found that SASP activated these cells to myofibroblasts in an autocrine and paracrine manner. This might be VEGF-mediated in the SASP medium, as the process can be prevented by nintedanib [90]. Chiu's [91] team confirmed that in the skin of SSc patients, the phenomena of EMT and fibroblast senescence were significantly increased, and the ccn2 in the SASP medium could induce the occurrence of EMT and promote the process of skin fibrosis (Fig 3).

### 2.3 Autophagy, endoplasmic reticulum stress, and fibrosis

Senescent cells can be eliminated by autophagy to maintain cell homeostasis. In the study by Nakamura et al., run domain beclin-1 interaction and inhibition of autophagy by cysteine-rich domain-rich proteins in renal tissues were identified as one of the features of aging [92]. Defective autophagy in podocytes can promote aging and lead to progressive glomerulosclerosis [93]. Inhibition of autophagy can promote cell senescence induced by oncogenes [9, 94]. In patients with non-alcoholic steatohepatitis (NASH), defective autophagy of hepatic endothelial cells enhances the early inflammatory characteristics of NASH and the expression of endothelial mesenchymal factors promotes the upregulation of inflammatory pathways, including CCL2, CCL5, IL-6 and VCAM-1 expression [95]. CCL2 family chemokines promote the aging of autophagy-deficient liver [96], while other factors promote liver fibrosis to varying degrees. In addition, defective autophagy in the liver can activate Nrf2 and further regulate liver aging, leading to cirrhosis [96]. Reduced autophagy in the lungs of IPF patients can induce epithelial-mesenchymal transition, promote the deposition of extracellular matrix in lung fibroblasts, and accelerate the fibrosis process [97-99]. In the study by Larson-Casey JL et al., AKT1-mediated mitophagy helps to resist alveolar macrophage apoptosis and delay the progression of pulmonary fibrosis [100]. Moreover, cellular senescence caused by impaired autophagy is also associated with cardiac dysfunction. Heart-specific deletion of Atg5 leads to impaired autophagy in cardiomyocytes and causes age-related cardiomyopathy [101].

Endoplasmic reticulum (ER) is an important component of vesicle transport and protein synthesis [102], and its function cannot be underestimated. In response to ER stress, eukaryotic cells generate the "unfolded protein response" (UPR) to adapt to the ER nuclear signaling system [103, 104] to cope with ER stress or to execute cell death. However, when the tolerance limit of UPR is exceeded, ER stress can lead to different degrees of damage to various tissues and organs, and then lead to fibrosis [105-107]. ER stress is associated with pulmonary fibrosis by regulating alveolar epithelial cell apoptosis, epithelial-mesenchymal transition, myo-fibroblast differentiation, and M2 macrophage polarization [108-110].In the report by Torres-Gonzalez et al. [111], ER stress markers were increased in type II alveolar epithelial cells in aged IPF mice compared with young IPF mice, thus supporting a link between ER stress and aging and fibrosis. In senescent cardiomyocytes, ER stress can lead to cardiac remodeling and systolic dysfunction [112]. ER stress can activate hepatic stellate cells, leading to massive ECM deposition and promoting liver fibrosis. Moreover, in primary biliary cirrhosis (PBC), ER stress can lead to autophagy dysregulation and cellular senescence of biliary epithelial cells, and abnormal expression of mitochondrial antigens leads to cirrhosis [113].

### 2.4 Mitochondrial dysfunction and fibrosis

One of the typical features of aging is mitochondrial dysfunction and stress, which leads to altered cellular metabolic pathways, increased ROS production, and decreased autophagy, leading to organ damage. A number of studies have revealed that mitochondrial dysfunction causes metabolic pathways such as glucose and amino acids to be carried out outside the mitochondria, which is one of the mechanisms leading to liver cirrhosis [114, 115]. Mitochondrial dysfunction not only leads to oxidative stress and inflammation cascade caused by increased ROS in renal tubular cells to accelerate the progression of renal fibrosis [116, 117], but also the decrease of mitophagy promotes renal fibrosis after renal ischemia-reperfusion [118-120]. PINK1 is a kinase associated with age-related neurodegenerative diseases [121]. Dysfunctional mitochondria in AT2 in IPF and aging lungs are associated with low expression of PTEN-induced putative kinase 1, a regulator of mitochondrial homeostasis [122-124]. Mitochondrial accumulation has been found in senescent lung cells from IPF patients, most likely due to mitochondrial dysfunction leading to reduced autophagy. At the same time, mitochondrial function and structure are also regulated by endoplasmic reticulum and autophagy. Endoplasmic reticulum and autophagy dysfunction lead to mitochondrial dysfunction and aggravate fibrosis process through a cascade reaction.

**Figure 4.**
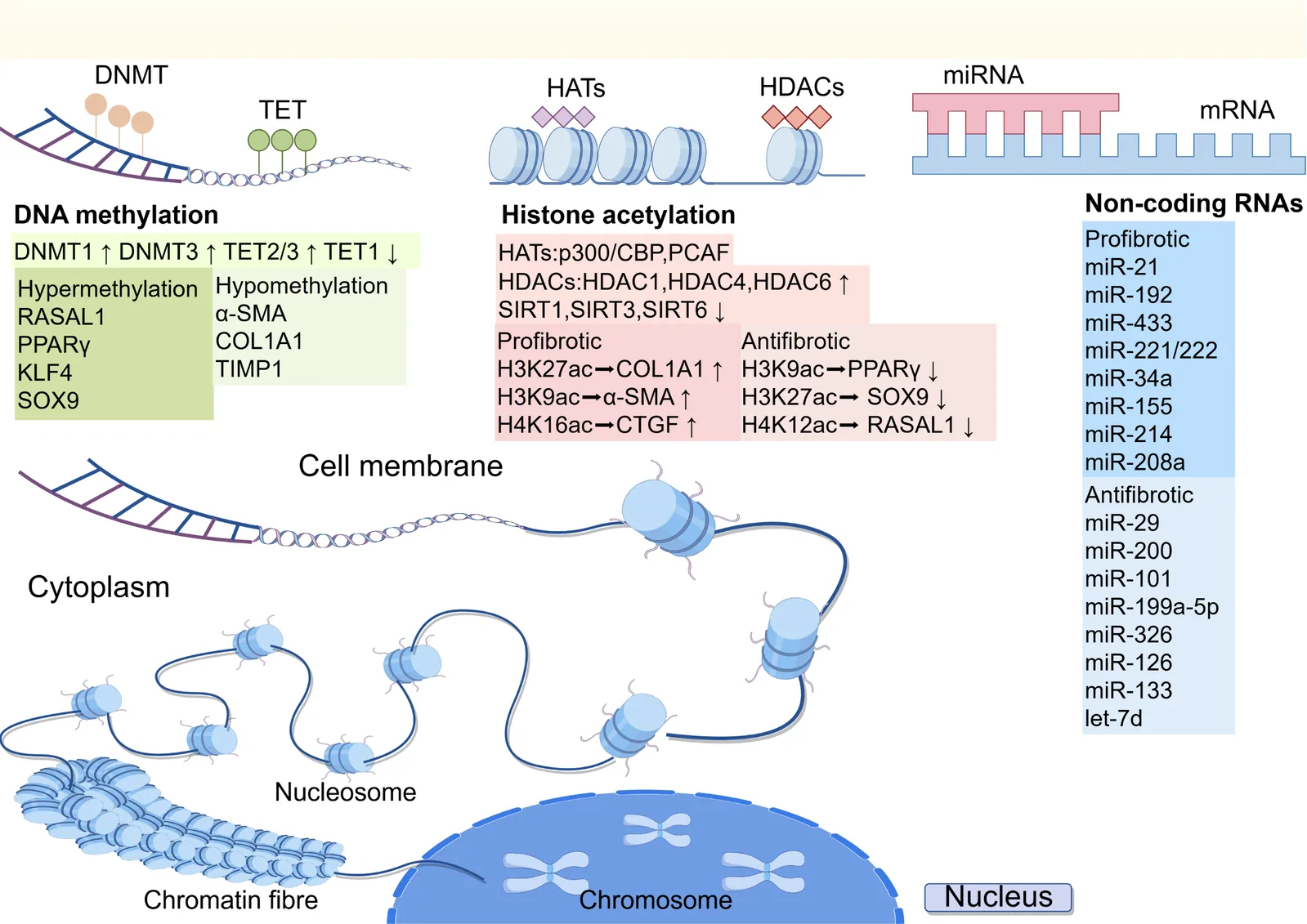
**Epigenetic mechanisms**. The three epigenetic mechanisms of DNA methylation, histone modifications and non-coding RNAs play a crucial role in the process of organ fibrosis.

### 2.5 Epigenetic alterations and fibrosis

Epigenetics refers to the control of gene expression through DNA methylation, histone acetylation modification, and interaction with transcription factors and non-coding RNA without changing DNA sequence [261, 124, 125]. More and more studies have proved that epigenetic changes are one of the important drivers of cellular senescence [125-127]. DNA methylation is a reaction in which a methyl group is added at the carbon 5 site (CpG site) of a cytosine residue [128], which usually results in the loss of gene expression due to the CpG being located in the promoter region [129]. Liu et al. [130] found differential methylation sites in renal tubules in the study of cytosine methylation in renal fibrosis, and found significant methylation differences in a number of known renal fibrosis genes, such as TGF-β receptor 3 (TGFBR3), SMAD3 and SMAD6 [131]. There are also different sites and different degrees of DNA methylation in the whole blood of patients with renal fibrosis [132]. P16 methylation down-regulates the expression of p16 mRNA and protein in lung tissue, which is one of the important mechanisms leading to pulmonary fibrosis [133, 134]. Zhao et al. [135] used reverse DNA methylation drugs to demethylation P16 gene to inhibit the growth of lung fibroblasts in vitro and successfully delay the process of pulmonary fibrosis [136]. Studies have found that abnormal DNA methylation can induce the proliferation of hepatic stellate cells and promote liver fibrosis in chronic liver diseases caused by various causes [137, 138]. In addition, DNA methylation of important genes can lead to atherosclerosis [139], and studies have shown that patients with coronary atherosclerosis are associated with DNA hypermethylation [140]. Furthermore, in IPF patients, anti-fibrotic genes show hypermethylation [141]. DNA methylation plays an important role in the heterogeneity of fibroblasts from IPF patients and promotes the progression of IPF [142]. Most often, histone acetylation promotes DNA transcription [143, 144]. In the process of fibrosis, histone acetylation participates in the process of fibrosis by promoting EMT, activating myofibroblasts [145], secretion of pro-inflammatory factors [146, 147] and pro-fibrotic factors [148]. Histone acetylation can directly regulate the gene expression of inflammatory cytokines such as IL-6 and fibronectin, thus affecting the progression of pulmonary fibrosis [149, 150]. The role of non-coding RNA (ncRNA) in fibrosis cannot be underestimated, and microRNA (miRNA) seems to be most closely related to fibrosis. Micrornas are involved in the epithelial-mesenchymal transition and fibroblast-myofibroblast transition involved in the process of pulmonary fibrosis [151], among which miR-155 and miR-21 promote fibrosis, while miR-107, mir-126, miR-140 and miR-511 have anti-fibrosis effects [152]. In addition, miR-9-5p affects chronic kidney injury and renal fibrosis by stimulating the reprogramming of metabolic imbalance and mitochondrial dysfunction [153], while the expression of miR-21, miR-192, miR-214 and let7 family can promote the process of renal fibrosis [154]. miR-378 is involved in the development of liver inflammation and fibrosis by enhancing the activity of the NF-κB-TNFα axis [155]. miR-144-3p can enhance the proliferation, migration and collagen synthesis of cardiac fibroblasts [156]. Cardiomyocyte specific overexpression of miR-328 can promote collagen deposition and induce cardiac fibrosis by inducing the TGF-β pathway [157].

Cell senescence and fibrosis cascade in layers. Excessive accumulation of senescent cells promotes the process of fibrosis, while fibrosis of organs and tissues accelerates the aging of normal cells. Furthermore, aging-related characteristics such as DNA damage, telomere dysfunction, SASP secretion, mitochondrial and endoplasmic reticulum dysfunction, and autophagy dysfunction interact with each other to promote cell senescence and tissue fibrosis (Fig 4).

## 3. Senescent clearance-based strategies for the treatment of fibrosis

Therapies to improve or delay cell senescence properties are important strategies to delay aging. At present, a variety of interventions have been developed to reduce aging or treat aging-related diseases, including lifestyle intervention, gene therapy, cell therapy, immuno-modulation, and drug intervention [158, 159].

### 3.1 The role of lifestyle in delaying cell senescence

Research reports that a good lifestyle is an effective treatment for anti-aging. Guagnano et al. reported that a diet low in oil and sugar and moderate exercise can significantly delay the progression of NAFLD [160]. Multiple studies have proved that dietary management and moderate exercise can alleviate tissue fibrosis [161-163]. Foods rich in vitamins, polyphenols and antioxidants exhibit powerful antioxidants and anti-inflammatory effects, protecting DNA from damage and delaying cell senescence [164]. In the study by McAlpine et al., adequate sleep was found to regulate the epigenome of hematopoietic stem cells (HSCS) and other progenitor cells, suppress inflammatory output, and maintain clonal diversity to slow down cellular aging [166, 171]. Aerobic exercises such as jogging and swimming can improve mitochondrial function and reduce chronic inflammation. Resistance training such as weightlifting can enhance antioxidant defense and cellular metabolism. These are all beneficial for preventing cell aging and improving cell functions [167].However,toxic components in cigarette smoke, including ROS and pro-inflammatory substances, can directly cause lung tissue damage and lung cell senescence, and induce chronic inflammation leading to pulmonary fibrosis [168]. Alcohol-induced oxidative stress and inflammation play an important role in the process of liver fibrosis [169]. Therefore, restricting food calories and maintaining good living habits such as appropriate exercise can significantly delay the process of cell aging and fibrosis.

### 3.2 The role of gene therapy in the elimination of senescent cells in the treatment of fibrosis

Gene therapy modulates the expression of a damaged gene by modulating the gene, either alone or in combination with cell therapy. Scientists have developed gene editing technologies such as zinc finger ribozymes (ZFNS) and transcription active effector ribozymes (TALENS), as well as CRISPR-Cas9. Among them, clustered regularly interspaced short palindromic repeats CRISPR-associated protein 9 (CRISPR-Cas9) can target multiple genes simultaneously for precise DNA editing. CRISPR-Cas9 gene therapy can accurately modify key genes such as ADAM9, GP96 and FAS in liver fibrosis, and effectively treat liver fibrosis [172]. Choi's team [173] edited amniotic mesenchymal stem cells (AMM/I) by TALENS technology, and the ILL-10 secreted by them has anti-inflammatory and anti-fibrosis effects, which can improve liver fibrosis. Researchers have targeted exogenous miR-29A to the kidney based on gene editing technology [174] to improve renal fibrosis. Ji et al. [175] used gene editing technology to target the muscle satellite cell-derived RVG-MIR-23-A/27A/26A cluster to the kidney to inhibit the expression of SASP related genes and significantly improve renal fibrosis. Gene therapy has brought a new and effective treatment for age-related fibrosis diseases. However, the precise targeting of senescent cells and the off-target effects of gene editing are problems that researchers have to consider. In addition, the ethical issues of gene editing technology have been controversial.

**Figure 5.**
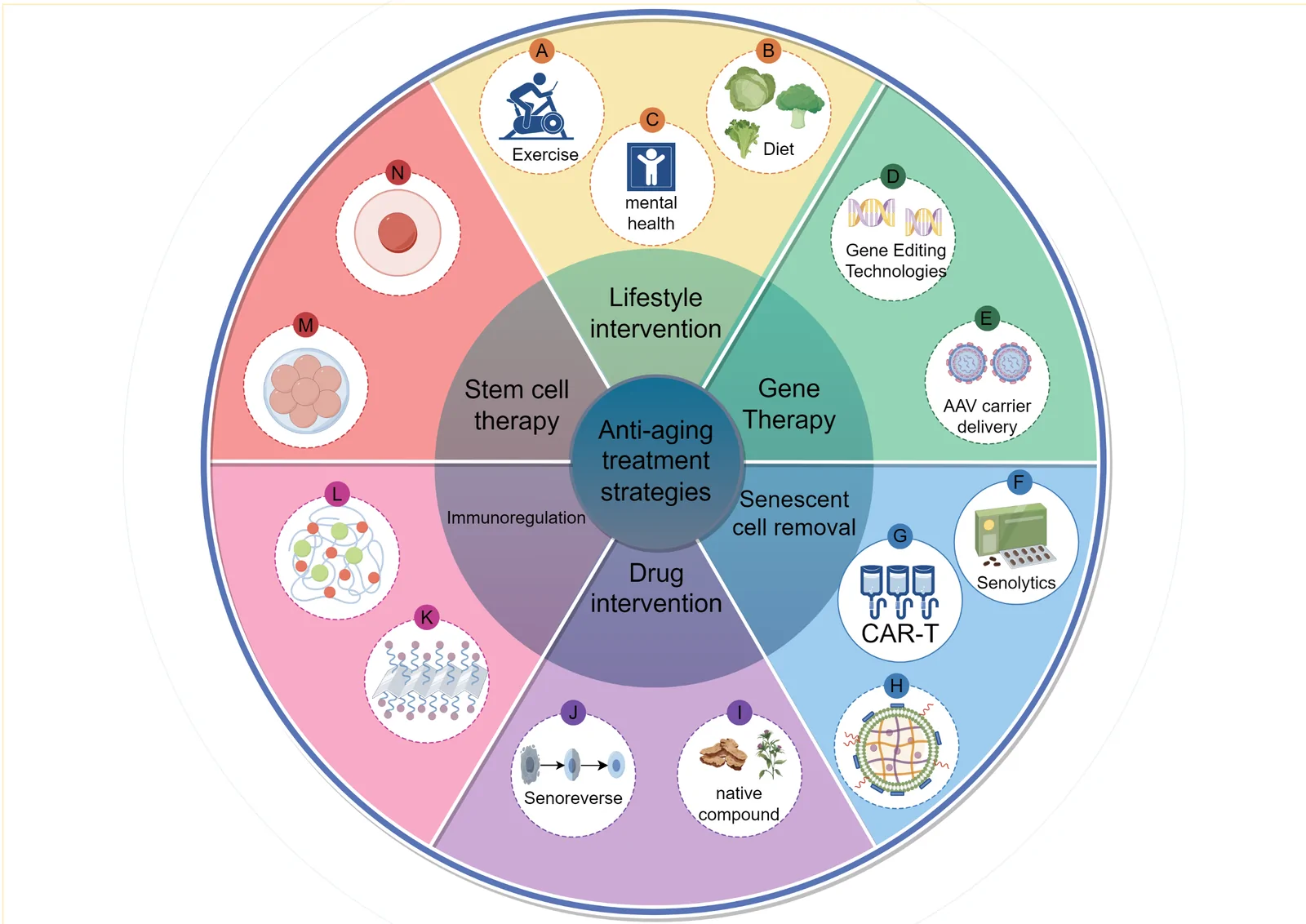
**Anti-aging strategy map**. At present, anti-aging strategies are mainly lifestyle intervention, gene therapy, cell therapy such as stem cell therapy, immunotherapy and drug therapy, so as to achieve the purpose of eliminating senescent cells to treat fibrotic diseases. Lifestyle intervention included moderate exercise, diet and mental health. Gene therapy can be used to introduce the target gene into the body through gene editing and different vectors. Cell therapy eliminates the harmful effects of senescent cells by injecting stem cells to reduce the overall proportion of senescent cells. Medical treatment included natural anti-aging drugs as well as clinically common cytotoxic drugs such as dasatinib (D) and quercetin (Q).

### 3.3 The role of cell therapy in the removal of senescent cells in the treatment of fibrosis

Stem cell therapy and immune cell therapy have made breakthroughs in the treatment of fibrotic diseases by eliminating senescent cells. Stem cells have strong differentiation potential and are essential for tissue damage repair. Adult stem cells, such as mesenchymal stem cells (MSCs), have been the most studied therapy for aging-induced fibrosis [176, 177]. Nelly et al. [178]successfully applied MSCs to delay skin and lung fibrosis, confirming that MSCS are an effective method to eliminate senescent cells and treat fibrotic diseases. In addition, MSCs can regulate the expression of TGF-β fibroblast factor and inhibit fibroblast proliferation and collagen deposition, thereby reducing tissue fibrosis [179, 180]. Researchers have delayed the progression of pulmonary fibrosis by effectively eliminating senescent interstitial cells by bronchial injection of adipose-derived mesenchymal stem cells [181]. Studies have shown that injection of adipose-derived mesenchymal stem cells into renal blood vessels can significantly improve renal fibrosis [182, 183]. Chiu et al. used autologous hematopoietic stem cell transplantation (aHSCT) to treat systemic sclerosis and found that the skin vascular aging and fibrosis markers of the patients decreased. It indicates that one of the effect mechanisms of autologous stem cell transplantation may be the reduction of senescent cells and SASP [184]. However, due to the limited source of stem cells, individual differences and rejection reactions, the consistency of treatment effect is affected, so further experimental and clinical observation is needed.

**Table 1:** Summary of anti-aging drugs.

Class	Drugs
**BCL-2 family inhibitors**	ABT-263 (Navitoclax)
	ABT-737
	A-1155463
	A-1331852
	EF24
	Fisetin
**HSP90 inhibitors**	17-DMAG (Alvespimycin)
	17-AAG (Tanespimycin)
	Geldanamycin
	Ganetespib
	Panobinostat
**p53 pathway targeting compounds**	FOXO4-DRI
	UBX0101
	RG7112 (RO5045337)
	P5091
**Natural products and their analogues**	Flavonoid (Quercetin, Amentoflavone)
	Apigenin
	rutin
	curcumin
	Resveratrol
	TP (tea polyphenol)
**Cardiac glycosides**	Ouabain
	Digoxin
	Proscillaridin A
	Digitalis purpurea
**NF-κB pathway inhibitors**	Metformin
	JSH-23
	SR12343
	8K-NBD
	Rapamycin
**Others**	Dasatinib + Quercetin
	PAI1 inhibitor(TM5275)

Chimeric T cell antigen receptor (CAR-T) treatment as a major breakthrough in the field of cellular immunotherapy, brought new treatments for many refractory diseases. The Janina Auth team used CD19-targeted CAR-T cells to treat systemic sclerosis and found that the patients had decreased antinuclear antibodies and systemic sclerosis specific autoantibodies, preventing the further progression of fibrotic diseases [185]. The team of Haig [186] used chimeric antigen receptor T-cell therapy (CAR-T) therapy to significantly reduce cardiac fibrosis in mice. Recently, researchers used M-UPA-M-28ZCAR-T therapy to specifically eliminate senescent hepatocytes, greatly improving liver fibrosis. Moreover, injection of low doses of M.19M.28Z or UT CAR-T cells can target depletion of senescent cells in the lung to improve pulmonary fibrosis [187]. Yang et al. [188] used NK2D-CART cells to effectively eliminate senescent cells and delay the process of pulmonary fibrosis. At the same time, Hnkg2d-CART cells could significantly eliminate senescent cells in liver and kidney organs and successfully improve liver and kidney diseases caused by cell senescence. Although CAR-T therapy has an excellent performance in eliminating senescent cells in fibrotic diseases, its injection dose is different from person to person, and it is prone to cause adverse reactions such as cytokine release syndrome (CRS) and neurotoxicity, and the treatment cost is high. As an emerging therapy, its long-term effect and potential risks still need to be further studied (Fig 5).

### 3.4 Application of anti-aging drugs in tissue fibrosis

The main role of anti-aging drugs is to eliminate or delay the adverse effects of cellular senescence, and thus the pathological processes associated with aging and fibrosis. At present, anti-aging drugs are mainly used to treat fibrosis by selectively eliminating senescent cells (senolytic) or blocking SASP. First-generation senolytics, such as dasatinib (D) and quercetin (Q), have been tested and shown promising efficacy in various preclinical models of aging and fibrotic diseases. In the mouse model of IPF, D+Q treatment improved lung compliance [189] The latest clinical research shows that the senolytic therapy represented by D+Q has achieved promising efficacy and good safety in the treatment of patients with IPF. Schafer et al. [190] demonstrated that D+Q treatment could eliminate senescent fibroblasts and delay pulmonary fibrosis. O 'Reilly treated senescent SSc cells with D&Q and found that SASP factor, especially IL-6, decreased significantly, and the corresponding senescent cells decreased significantly [191].Triana-Martinez et al. [192] demonstrated that cardiotonic agents such as digoxin eliminate senescent cells to eliminate pulmonary fibrosis. Navitoclax drugs reduce aging and improve cardiac function by attenuating profibrotic SASP and promoting angiogenesis [193]. Inhibition of SASP secretion is one of the strategies of some anti-aging drugs, and the second is to target the pathway related to SASP expression. A number of studies have shown [194] that anti-aging drugs procyanidins C1 (PCC1) and astaxanthin can improve IPF through ROS-dependent mitochondrial signaling pathways in AT2. In renal fibrosis, especially chronic renal fibrosis caused by diabetes, metformin has been shown to inhibit the NF-κB signaling pathway in fibroblasts, macrophages and senescent endothelial cells to delay the process of renal fibrosis [195]. The HSP90 inhibitor avispiamycin reduces atherosclerosis by inhibiting NF-κB and STAT signaling pathways [196]. Baar's group [197] discovered FOXO4-D-Retro-Inverso (FOXO4-DRI) peptide, which blocks the interaction between FOXO4 and p53, thereby inducing apoptosis in senescent cells and promoting the restoration of normal cellular homeostasis in fibrosis. Many clinical drugs have been confirmed to have the effect of clearing senescent cells. For example, rapamycin can improve cell senescence and delay the process of fibrosis by inhibiting the mTOR pathway in SASP [198]. In the study of Johmura et al. [199], it was found that glutaminase-1 inhibitors can eliminate senescent cells and improve renal fibrosis by inhibiting the decomposition of glutamine. More and more studies have shown that natural compounds such as quercetin, flavonoids such as taxifolin [200], rutin [201], anthocyanins, etc., can improve cell senescence to varying degrees by inhibiting different factors in SASP such as NF-κB, MT and JAS-STAT signaling pathways [202, 203]. Moreover, anti-aging drugs such as HSP90 inhibitors, BCL-XL inhibitors, A1331852 and A1155463 have been tested in different fibrosis models such as pulmonary fibrosis and have initially shown to reduce the development of the disease after anti-aging [204]. Anti-aging drugs have been studied in natural compounds, kinase inhibitors, Bcl-2 family inhibitors/Bcl-2 homolog 3 (BH3) mimetics, MDM2/p53 interaction inhibitors, Hsp90 inhibitors, and p53 binding inhibitors [205]. Based on the summary by Kim and Lei et al. [206], we have summarized the classification and drugs of anti-aging medications in the treatment of fibrotic diseases (Table 1), as well as the clinical trial stages of some of these drugs (Table 2). In summary, anti-aging drugs have achieved promising results in both in vitro and preclinical studies of fibrosis.

**Table 2:** Senolytic indication and clinical trials.

Senolytic	Indication	Clinical trials
**Dasatinib + Quercetin**	IPF	Phase 1 completed, Phase 2 in process
	Diabetic chronic kidney disease	Phase 2 completed
	Alzheimer’s disease	Phase 1 completed, Phase 2 in process
**Fisetin**	IPF	Phase 1 completed, Phase 2 in process
	Chronic kidney disease	Phase 1 completed, Phase 2 in process
	Alzheimer’s Disease	Phase 2 completed
	Osteoarthritis	Phase 1 completed, Phase 2 in process
	Age-related osteoporosis	Phase 2 completed
	Complications due to COVID-19	Phase 1 completed, Phase 2 in process
**ABT-263**	Myelofibrosis	Phase 2/3 in process
**UBX0101**	Osteoarthritis	Phase 2 completed
**Rapamycin**	Alzheimer’s Disease	Phase 1 completed
	Amyotrophic Lateral Sclerosis	Phase 2 completed

### 3.5 The disadvantages of anti-aging drugs in organ fibrosis

With the continuous discovery of anti-aging drugs and the increasing application of them in various aging-related fibrotic diseases, their potential adverse reactions have received more attention. For example, compounds with senolytic activity such as Bcl-2 inhibitors ABT-263 and ABT-737 face significant challenges in clinical application transformation because they can induce thrombocytopenia, neutropenia and off-target effects [207]. Natural compounds such as quercetin and flavonoids are often accompanied by problems such as low bioavailability, complex pharmacokinetic characteristics, and large variability in drug efficacy among individuals [208]. Other senolytic drugs can act on core pathways such as TGF-β and mTOR, and their extensive multi-target effects may unexpectedly disrupt normal physiological homeostasis [209]. For example, excessive clearance of specific senescent cells may weaken the tissue's repair ability or trigger an inflammatory storm; mTOR inhibitors may induce immunosuppression or metabolic disorders [210].

Due to the significant heterogeneity among individual patients, it will affect the distribution, metabolism and clearance of drugs, increase the risk of drug accumulation and drug interactions, and amplify the susceptibility and severity of potential adverse drug reactions. Furthermore, the current evidence for the effectiveness of most anti-aging drugs stems from preclinical models or short-term small-scale human trials. The lack of long-term, large-scale randomized controlled trials (RCTS) data for the frailty leads to significant uncertainties in the safety of their long-term use, such as potential carcinogenicity and long-term effects on neurocognitive function [211, 212]. Therefore, we still need to further explore safer and more effective anti-aging drugs, and conduct more and larger-scale clinical studies at the same time to observe the long-term safety of anti-aging drugs.

## 4. Application of emerging omics technologies in senescence and fibrotic diseases

With the rapid development of omics technology, genomics, transcriptomics, metabolomics, proteomics and other technologies play an important role in the pathogenesis, diagnosis and treatment of aging and fibrosis diseases.

### 4.1 Application of genomics in senescence and fibrotic diseases

Genomic technology reveals the molecular mechanism of cell senescence in fibrosis by identifying genes and pathways related to cell senescence in organ fibrosis, such as p16INK4, p21Sdi1/Cip1/WAF1, etc. [213]. The development of genome and epigenome editing tools has allowed the editing of specific genes in fibrosis to treat fibrosis. In addition, genomics has an important application in the development of drugs for the treatment of fibrosis. Through high-throughput screening and functional genomics tools, new drug targets are discovered to treat fibrotic diseases [214].

### 4.2 Application of transcriptomics in senescence and fibrotic diseases

Single-cell sequencing technology can not only reveal specific gene expression patterns, cellular heterogeneity and complexity, and dynamic changes in different cell subsets in fibrosis, but also reveal a variety of signaling pathways related to aging and fibrosis, helping people to further understand their molecular mechanisms. Lee et al. [215] successfully used single-cell RNA sequencing technology (snRNA-seq) to delineate the functional changes of fibroblasts during aging and found that torsin-1A interacting protein 1 was abnormally expressed in aging fibroblasts. Nelke et al. [216] used snRNA-seq to identify a specific group of fibro-adipose progenitor cells that exhibit key features of aging, including proinflammatory phenotypes, p21 expression, increased β-galactosidase activity, and the involvement of other aging pathways. The rise of spatial transcriptomics (ST) allows people to identify different cell types and their interactions on tissue sections, and discover the spatial variation of different gene expression and its distribution characteristics [217]. By integrating ST with scRNA-seq data, Franzen et al. [218] revealed that fibroblasts and alveolar macrophages in IPF lungs exhibit significant gene expression changes, including, but not limited to, upregulation of genes such as FNDC1, COL10A1, and THY1. In IPF, TGF-β signaling predominates in the fibrotic niche. By integrating ST, scRNA-seq, genetic lineage tracing and other results, researchers found the cell differentiation and invasion process of lung fibroblasts from IPF patients, and determined that SFRP1 regulates TGFβ1-induced fibroblast invasion and RHOA pathway activity [219]. Shi et al. conducted scRNA-seq on the skin of patients with systemic sclerosis and found that the characteristic spectrum of aging genes was significantly elevated, which was related to fibrosis [220].In the study of liver fibrosis, comprehensive analysis of ST and scRNA-seq data has found that hepatic stellate cells (HSC) are the main source of myofibroblasts in liver fibrosis, and senescent HSC is an effective target for anti-fibrosis therapy [221]. Studies have found that SRSF1 can reverse the cellular aging process through transcriptome reprogramming, which provides a new direction for the development of anti-aging and anti-fibrosis drugs. ST combined with scRNA-seq can not only provide accurate spatial information of fibrotic lesions to predict disease progression, but also help to analyze the mechanism of effective drugs in fibrotic diseases and accelerate the discovery of new drugs by predicting drug action pathways. By integrating ST data from non-alcoholic fatty liver disease (NAFLD) patients, gene expression differences between patients with stable fibrosis and those with progressive fibrosis were evaluated, and PON1 and FLNA positive patients were found to have rapid disease progression and poor prognosis [222].

### 4.3 Application of metabolomics in senescence and fibrotic diseases

**Figure 6.**
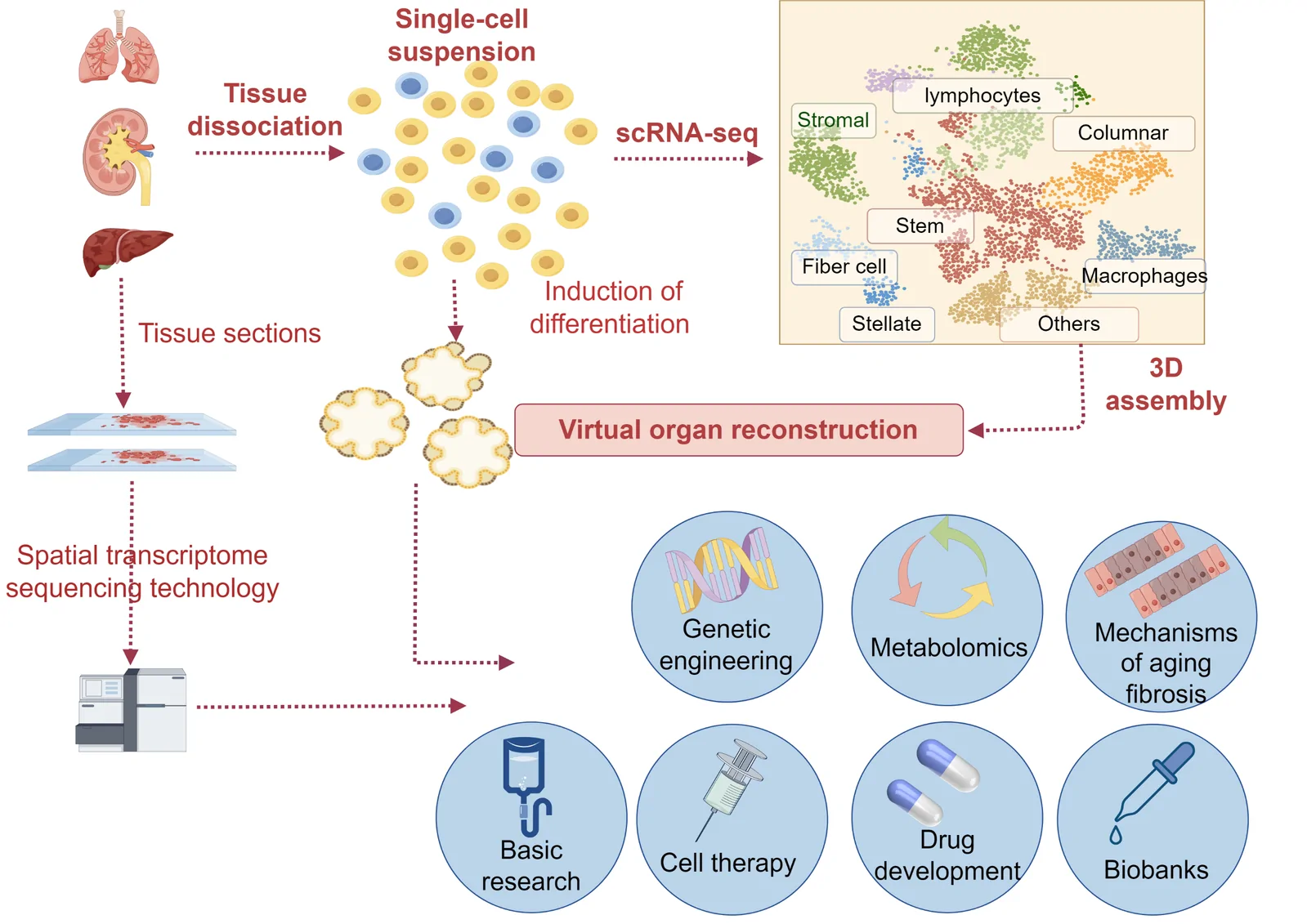
**Application of emerging multi-omics techniques in aging and fibrosis**. The obtained clinical tissues can be cultured as organoids or processed into single-cell suspension for single-cell sequencing. Organoids can be used to observe the pathological process of fibro-related diseases or perform organoid chip to screen effective and safe anti-aging drugs for high-throughput delay or reverse fibrotic diseases. Different clinical tissues obtained at the same time can be tested by genomics, proteomics, and metabolomics, which will provide new diagnostic markers and new treatment strategies for aging fibrosis-related diseases.

Metabolic reprogramming and metabolic dysregulation are prominent features of fibrosis [223]. Studies have proved that proline derived from the decomposition of glutamine is a key component of collagen and ECM [224]. According to the metabolic analysis of plasma in IPF patients, TGF-β1 up-regulates the expression of glutaminase by activating Smad3 and p38-MAPKdependent signaling to accelerate the decomposition of glutamine into proline and produce collagen [225]. Researchers have conducted lipid metabolomics analysis of serum and bronchoalveolar lavage fluid of IPF patients and found that abnormal sphingolipid metabolism, especially sphingosine 1-phosphate (S1P) and lysophosphatidic acid (LPA), are important parts of the pathogenesis of IPF [226]. Metabolic alterations of amino acids such as glycine and arginine can eventually lead to organ fibrosis by triggering abnormal collagen synthesis and dysregulation of airway remodeling. These metabolomic alterations provide a new perspective for understanding the pathogenesis of IPF [227]. Through metabolomics analysis of the serum of patients with NAFLD, Caussy et al. successfully identified lipid metabolite markers related to organ fibrotic activity, as well as eicosanid-like metabolite markers that can predict the progression and clinical stage of fibrosis [236]. Researchers have successfully applied metabolomics to the clinical study of liver fibrosis: Through metabolomic analysis of the blood and urine of patients with liver fibrosis, multiple biomarkers predicting complications of liver fibrosis, such as PRO-C3 and ELF™, have been identified. The diagnostic strategies established based on these findings have achieved early diagnosis and intervention of the disease, significantly improved the prognosis of patients, and demonstrated the effective transformation of metabolomics into clinical practice [237]. Through metabolomics analysis of blood and urine, researchers discovered alterations in multiple key metabolic pathways such as metabolites of tryptophan, the citric acid cycle, and the urea cycle in CKD. And potential biomarkers and intervention targets such as urinary protein and kynurenine were identified. These findings were successfully translated into clinical applications, achieving early diagnosis and precise intervention of CKD, thereby significantly improving the clinical prognosis of patients [232, 233] (Fig. 6).

### 4.4 Application of proteomics in senescence and fibrotic diseases

Proteomics has great potential in clinical diagnosis and antibody characterization through protein identification, quantification and functional analysis of signaling pathways and networks [234]. In addition, it can study the interaction between proteins with unknown structure and high-affinity ligand molecules, and provide evidence for the development of new anti-aging and anti-fibrosis drugs [235]. Marko-Varga's group used proteomics to elucidate that TGF-β induces not only α-SMA but also a whole set of actinin-related proteins involved in the formation of stress fibers, cell contractility, and cell spreading that characterize myofibroblast phenotypes [236]. Through proteomics studies, researchers have not only identified proteins such as EGFR and their downstream signaling pathways as key targets for renal fibrosis, but also discovered that the natural product ascofuranone can be used as a novel treatment strategy for fibrotic nephropathy. Urinary proteomics in CKD patients confirms that proteinuria is associated with fibrogenesis and progression of renal fibrosis through direct EMT [237] or albumin-induced cytokine release from renal tubular epithelial cells [238]. Proteomic analysis of myocardial fibrosis before and after medication was performed by staff, and it was clear that natural products can inhibit oxidative stress and regulate aging through the p53 signaling pathway to reduce myocardial fibrosis [239]. A proteomic analysis of plasma from patients with advanced IPF by Sivakumar's group [240] identified several diagnostic and prognostic markers. In addition, proteomic detection of sweat [241], serum [242], and urine [243] has found defects in protein processing mechanisms caused by endoplasmic reticulum stress, the occurrence of UPR, and the pathogenesis of protein hydrolysis in patients with cystic fibrosis.

The development of multi-omics technology allows people to comprehensively analyze the molecular mechanism of fibrosis during aging through different combinations, identify more potential biomarkers related to aging fibrosis, and provide an important scientific basis for the development of new treatment strategies. Such as the first reported drug combination of dasatinib (D) and quercetin (Q), an anti-aging drug, was discovered from a hypothesis-driven, bioinformatics based study.

## 5. Applications of emerging model techniques in senescence and fibrotic diseases

### 5.1 Applications of organoids in senescence and fibrotic diseases

Organoids are three-dimensional structures derived from patients' tissues or cells (such as adult stem cells or pluripotent stem cells), which are highly human. In addition, organoids have a short culture cycle, a high success rate, and can be established quickly. Organoids offer significant advantages over 2D cell cultures and animal models in terms of structural complexity, cellular heterogeneity, and physiological relevance. It can better simulate the development, function and disease state of human organs, so as to improve the reliability and predictability of research results [244]. Researchers have generated fibroblast-dependent alveolar organoids from human pluripotent stem cells and primary human fetal lung fibroblasts (HFLF) [245] to enable human alveolar epithelial cells and fibroblasts to interact in pulmonary fibrosis. Senescence of epithelial cells in AT2 cells and the abnormally differentiated intermediate state AT2-AT1 cells during IPF progression [246], as well as fibroblast activation, organoid contraction, and extracellular matrix accumulation were found. In addition, ALK5 inhibitors were screened to improve the fibrogenic phenotype [247]. Recent studies have shown that inhibition of hypoxia-inducible factor 2(HIF2) in IPF lung organoids promotes the differentiation of epithelial progenitor cells into AT2 epithelial cells. The in vitro platform provided by the organoid model validates that HIF2 inhibitors can regulate the phenotypic plasticity of alveolar epithelial cells and promote functional repair and maturation, which provides sufficient pre-clinical evidence for the treatment of fibrosis. Organoid models of liver fibrosis can be used not only as preclinical models to test antifibrotic therapies, but also to identify drug targets. For example, researchers have found that PDGFR tyrosine kinase inhibitors (imatinib, etc.) inhibit TGFβ signaling pathway and show anti-fibrosis effect [248]. In order to further study the mechanism and drug targets of liver fibrosis caused by primary sclerosing cholangitis (PSC), Chen et al. established multicellular organoids and successfully found that inhibition of TH17 differentiation and improvement of Th17-induced microenvironment have anti-fibrotic effects in PSC disease [249]. Moreover, aging-related organoid biobanks have been created to provide promising preclinical models for research and drug screening purposes.

### 5.2 Organ-chip applications in senescence and fibrosis

Organ-on-chip utilizes microfluidic technology to realize cell-to-cell interaction and environmental simulation by embedding cells (including organoids) into the chip. By combining various fibrotic Organoids and organ-on-a-chip technologies, organoids can not only simulate the pathological process of fibrosis more accurately, but also provide new tools for disease treatment and drug screening, and facilitate the dynamic study of disease mechanisms and drug effects. Since the groundbreaking 'breathing lung' was introduced [250], organoid chips have developed rapidly, especially for drug evaluation of pulmonary fibrosis diseases. Asmani et al. [251] successfully simulated progressive stiffness and contraction of alveolar tissue using pulmonary fibrosis organoid chips, and also evaluated the potential biomechanical mechanisms of the antifibrotic drugs pirfenidone and ndanib. Isabelle [252] team observed the dynamic process of tendon fibrosis by constructing a fluid human tendon organoid chip. On this basis, high-throughput screening of drugs that limit immune inflammatory response eliminates senescent cells to reduce muscle fibrosis. Furthermore, based on the construction of cardiac fibrosis organoids, researchers have tested the efficacy of advanced miRNA therapy, which successfully delayed the process of cardiac fibrosis and improved cardiac function [253]. Researchers have used the pulmonary fibrosis microarray to outline the key pathological processes of pulmonary fibrosis as well as the conditions during inhalation therapy [254]. The development of liver spheroid organoid chips has clarified the role of hepatocytes in lipid accumulation and hepatocyte senescence in liver fibrosis diseases such as NAFLD [255]. Furthermore, Cho [256] research team not only summarized the characteristics of NASH but also showed the process of new blood vessel formation in the liver organoid chip. By constructing multi-organ chips to simulate the interactions between different organs to study systemic diseases, complex physiological processes can be realized in a smaller space [257].Moreover, organ-chip supports multiple experiments at the same time, allowing large-scale drug screening and toxicity testing.

The advent of organoids and organ-on-a-chip is crucial for the identification of therapeutic targets and personalized treatment in precision medicine. These models are better for evaluating the safety and efficacy of anti-aging therapies for fibrotic diseases. Second, these models can better reflect the novel concomitant biomarkers in fibrotic disease progression or regression with cellular aging, thus supporting the design of non-invasive diagnostic tests.

## 6. Conclusions

In conclusion, the continuous accumulation of senescent cells can lead to fibrosis of tissues and organs. The development of multi-omics technology provides a strong guarantee for researchers to study the molecular mechanism, pathological process, diagnostic markers and treatment of fibrosis caused by cellular senescence. We look forward to using emerging technologies to discover more close connections between aging and fibrosis and verify specific molecular mechanisms. We also hope to discover more effective and safe anti-aging strategies through emerging technologies to delay the progression of fibrotic diseases. There are more and more ways to treat fibrosis with anti-aging cells, providing potential new therapeutic strategies for the treatment of fibrosis. Although the results of early clinical trials in fibrosis-related diseases are encouraging, a further in-depth understanding of how senescent cells, especially the factors contained in SASP, precisely spread fibrosis will help determine new therapeutic targets. Among them, the discovery and research of drugs provide safer and more effective means for the treatment of fibrotic diseases. However, the best anti-aging method for disease treatment remains unclear. The safety and clinical individual efficacy of anti-aging cell therapy intervention measures for fibrotic diseases remain to be proven and further experiments are still needed for verification. We also need larger sample size prospective cohort studies, efficacy evaluations of new intervention strategies for anti-aging drugs, and acquisition of long-term follow-up data, etc. Meanwhile, we should actively promote the transformation of existing discoveries into clinical practice. Such as diagnostic or prognostic biomarker detection methods based on research findings and promoting their standardization and validation; Design and implement therapeutic clinical trials based on mechanism exploration; The feasibility and strategies of integrating research results into existing clinical guidelines or diagnosis and treatment processes; Challenges and solutions for the practical application of related technologies (such as AI analysis models) in clinical scenarios, etc. The specific path to make the research results ultimately serve the clinical practice and improve the prognosis of patients is our ultimate goal.

The accumulation of senescent cells is a key factor driving the fibrosis of tissues and organs. New technologies such as multi-omics provide strong support for revealing the molecular mechanisms, pathological processes, and therapeutic strategies of this phenomenon. In the future, it is imperative to utilize emerging technologies to deeply verify the close connection and core mechanisms between senescence and fibrosis and discover safer and more effective anti-aging intervention methods to delay the progression of diseases. Anti-aging therapy has great potential as a treatment strategy for fibrosis, but its optimal intervention methods, safety, and individual efficacy still need to be verified through rigorous clinical trials and large-scale prospective cohort studies with long-term follow-up. At the same time, we should actively promote the transformation of existing discoveries into clinical practice. With the help of emerging technologies, our ultimate goal is to make research achievements ultimately serve clinical practice and improve patient prognosis.
